# Breast Cancer Screening Awareness and Associated Factors Among Saudi Females: A Cross-Sectional Study in Jeddah, Saudi Arabia (2024)

**DOI:** 10.7759/cureus.60337

**Published:** 2024-05-15

**Authors:** Ghala Yasin, Abeer A Subke

**Affiliations:** 1 Preventive Medicine, Saudi Ministry of Health, Jeddah, SAU

**Keywords:** saudi females, saudi arabia, screening, awareness, breast cancer

## Abstract

Background: Breast cancer (BC) represents a major health concern for women globally, including those in Saudi Arabia. Awareness and early detection through screening practices are vital measures in improving BC outcomes.

Objective: This study aims to assess the awareness and associated factors of BC screening among Saudi females in Jeddah, Saudi Arabia.

Methods: A cross-sectional study was conducted in 2024 among Saudi females aged 18 and above, residing in Jeddah. Data was collected using a structured questionnaire that included sections on demographic characteristics, Breast Cancer Awareness Measure (Breast CAM), knowledge, and factors influencing participation in screening programs. The collected data was analyzed using IBM SPSS Statistics.

Results: The study, consisting of 454 participants, found that half of them rarely or never checked their breasts and many were not confident about noticing any changes in their breasts. There was a strong awareness of the Ministry of Health's (MOH) BC screening program, but most were uncertain about the age at which screenings should start and end. Only a small percentage reported receiving an invitation for breast screening and an even smaller percentage have participated in the screening. Significant associations were found between higher awareness of BC signs, a monthly family income of over 20,000, and being medically free (p-value <0.05).

Conclusion: The study found a significant gap in BC awareness and screening practices among females in Jeddah, Saudi Arabia. This underlines the importance of targeted health education and awareness programs, as well as accessible and affordable screening services, to improve early detection and outcomes for BC.

## Introduction

Breast cancer (BC) is the most common cancer in women, with an annual incidence of more than 1.4 million cases worldwide, making it the second most prevalent cancer after colorectal cancer [1]. In 2018, there were 2.1 million incident cases of BC diagnosed globally, with an estimated 627,000 predicted deaths [2]. In 2018, the incidence of BC in Saudi Arabia was 29.7% among 17,522 cases [3]. BC screening has several benefits. Primarily, it is crucial for early detection, which can lead to successful treatment and improved survival rates. It includes various methods such as mammography, ultrasound, magnetic resonance imaging (MRI), clinical breast exams, and self-exams [1,3,4]. There is a need for early detection procedures and screening programs to identify people who may be at risk of BC since it poses a serious threat to women's health. Cancer screening is a crucial part of secondary prevention since it can result in an early diagnosis and a higher chance of successful cancer therapy. Programs for early identification and screening have made a substantial contribution to the decline in BC death rates [4]. However, numerous sociodemographic predictors have been known to influence women's participation in BC screening programs like age, religion, income, work status, awareness of BC, cancer-related beliefs, and accessibility to screening services [5-7].

In a 2022 study conducted in Canada, it was found that retired women who had a higher income and had access to a family doctor were more likely to receive regular BC screenings [7]. A nationwide cross-sectional study was conducted in 2022 in Saudi Arabia and demonstrated that 71% of participants had low BC awareness levels. Women who were unemployed were more likely to have low awareness about BC [8]. In a study conducted among 232 female students and faculties of Najran University, Saudi, 75.3% of research participants had "good" overall awareness of BC, whereas 94.3% had "poor" knowledge of the warning indicators of BC. The factors that predicted "good" overall knowledge were age, family history, educational attainment, and marital status [9]. Among the 465 women in Madinah, Saudi Arabia, 38.5% of women underwent breast self-examination and 27.7% underwent mammography. The majority of the women in the study and those who had never had a mammogram had very low levels of awareness about BC, especially when it came to the risk factors for the disease [10]. In Malaysia, 99.1% of the 447 women who were enrolled in a 2014 study knew about BC; moreover, only 50.1% of people were aware of mammography screening. Approximately 23.3% of women received clinical breast examinations, while only 13.2% took part in mammography screening. Among those women, predictors for mammography screening were those who had undergone a clinical breast examination (adjusted OR=17.58, 95% CI: 7.68-39.82), those in the 50-59 age range (adjusted OR=3.94, 95% CI: 1.61-9.66), and those 60 years of age and beyond (adjusted OR=6.91, 95% CI: 2.28-20.94) [11]. A similar cross-sectional study conducted among 250 Malaysian women found that while most participants (81.2%) had heard of BC, the majority were unaware of its symptoms, risk factors, and screening options such as mammography [12].

BC is a significant concern for public health in Saudi Arabia, particularly in Jeddah. However, there are knowledge gaps regarding the awareness and predictors of BC screening among Saudi females. Therefore, this study is conducted to investigate the associated factors and provide insights that can inform targeted interventions and policies to increase screening rates and reduce the burden of BC. By conducting this research, valuable contributions can be made to the existing knowledge on BC screening in the Saudi Arabian context, laying the foundation for future research and interventions.

## Materials and methods

Study design and population

This cross-sectional study was conducted in 2024 in Jeddah, a major city in the Western region of Saudi Arabia. The study included Saudi females, aged 18 or above, residing in Jeddah and willing to participate. Non-Saudi females and those residing outside of Jeddah were excluded. The study strived to recruit a diverse participant pool in terms of age, education level, socioeconomic status, and occupation.

Sampling and sample size

**Figure 1:**
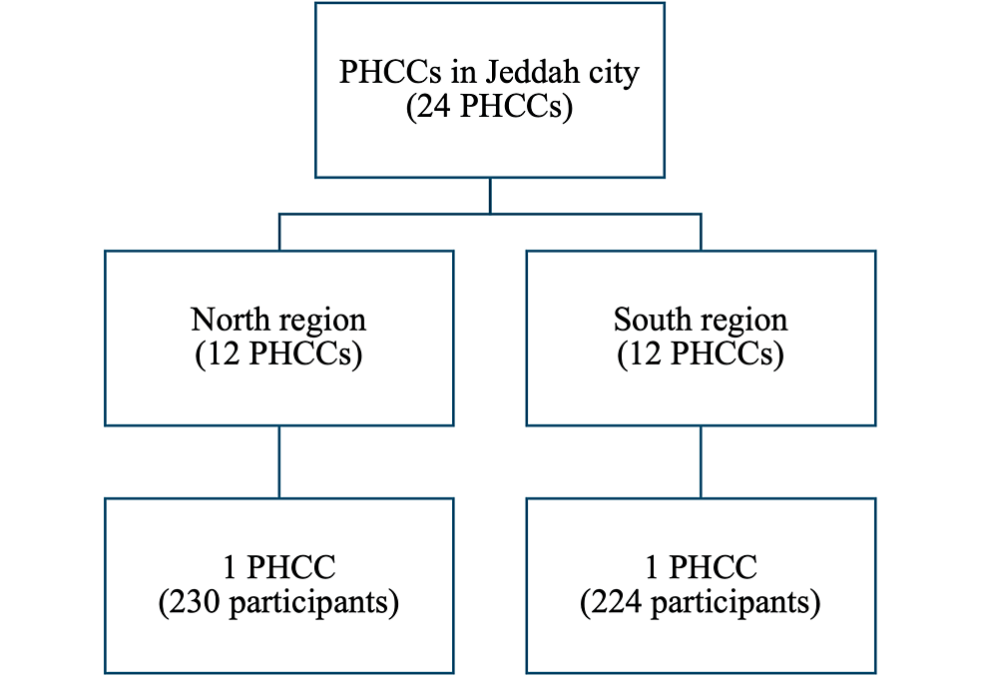
Flow chart of the sampling technique PHCCs, primary healthcare centers

The study employed a multi-stage sampling approach to enlist potential participants from the primary healthcare centers (PHCCs) in Jeddah. The first stage involved dividing Jeddah into two regions: North and South. The second stage involved obtaining a list of PHCCs from each region, with 12 PHCCs in each region. Two PHCCs were randomly selected from each region through simple random sampling. The third stage employed cluster sampling, which encompassed participant recruitment from the selected centers, focusing on those who met the inclusion criteria. This process aimed to ensure representation from both northern and southern parts of Jeddah, providing a comprehensive understanding of the awareness and predictors of BC screening among Saudi females in different city areas (Figure 1).

The study's estimated sample size was 385, calculated using an online sample size calculator (Raosoft.com, http://raosoft.com/) with a 5% margin of error, a 95% CI, and a 50% response distribution. However, the total sample size included in the study was 454 to account for missing data or potential exclusion.

Data sources and collection

Data was collected using a structured questionnaire with multiple-choice questions. The questionnaire, divided into sections, gathered data on demographic characteristics, awareness of BC screening, knowledge about BC, and factors influencing participation in screening programs. The questionnaire was designed based on relevant literature, including the Breast Cancer Awareness Measure (Breast CAM) [13]. The Breast CAM tool, consisting of a set of questions, was used to evaluate individuals' knowledge and understanding of BC, including risk factors, symptoms, and screening methods. In the Breast CAM, we assessed the level of awareness based on participants' answers. They were considered to have a sufficient level of awareness if they scored 60% or more, while a score below 60% indicated a low level of awareness. Trained research data collectors administered the questionnaire, ensuring the quality and accuracy of the data. Participants were informed about the study's objectives and confidentiality measures, and any concerns they raised were addressed.

Statistical analysis

The collected data was analyzed using IBM SPSS Statistics (version 29.0) (IBM Corp., Armonk, NY, USA). Categorical variables were presented as proportions, while numerical variables were presented as median ± interquartile range since the data were not normally distributed. The Chi-square and Fisher-Freeman-Halton exact tests were used for univariate analysis of categorical outcomes. A p-value less than 0.05 determined significance, and conclusions were drawn with a 95% CI.

## Results

This cross-sectional study consisted of a total of 454 participants. The median age of the participants was 36 years, with an interquartile range of 15 years. Meanwhile, the majority fell into the age group of 40 years or younger (68.9%). More than half of the participants were married (53.3%) and had children (57.7%). Most of the participants held a bachelor's degree or higher (64.1%) and were employed (56.4%). In terms of income, 43.8% of the participants reported a monthly family income of 10,000 Saudi Riyals or less. Regarding health conditions, 53.3% of the participants indicated they had no medical issues (Table 1)

**Table 1:** Demographic characteristics

n=454		N	%
Age (years)	≤40	313	68.9%
	41 - 60	129	28.4%
	>60	12	2.7%
Marital status	Single	153	33.7%
	Married	242	53.3%
	Divorced	45	9.9%
	Widowed	14	3.1%
Do you have any children?	No	192	42.3%
	Yes	262	57.7%
Educational status	No official education	30	6.6%
	High school or less	133	29.3%
	Bachelor's degree and above	291	64.1%
Employment status	No	198	43.6%
	Yes	256	56.4%
Monthly income of the family (Saudi Riyals)	≤10,000	199	43.8%
	10,001 – 20,000	198	43.6%
	>20,000	57	12.6%
Medically free		242	53.3%
Hypertension		58	12.8%
Diabetes mellitus		71	15.6%
Dyslipidemia		47	10.4%

When questioned about their breast-checking habits, 50.2% of the participants reported that they rarely or never checked their breasts. A significant number of participants were not confident (23.1%) or not very confident (32.8%) that they would notice a change in their breasts. Furthermore, 47.6% of the participants had never visited a doctor about a change they noticed in their breasts (Table 2).

**Table 2:** Participants’ practices and attitudes toward breast examination and screening BC, breast cancer;  MOH, Ministry of Health

n=454		N	%
How often do you check your breasts?	Rarely or never	228	50.2%
	At least once a week	20	4.4%
	At least once a month	67	14.8%
	At least once every six months	139	30.6%
Are you confident you would notice a change in your breasts?	Not at all confident	105	23.1%
	Not very confident	149	32.9%
	Fairly confident	95	20.9%
	Very confident	105	23.1%
Have you ever been to see a doctor about a change you have noticed in one of your breasts?	No	216	47.6%
	Yes	112	24.7%
	Never noticed a change in one of my breasts	126	27.7%
As far as you are aware, is there a MOH's BC screening program?	No	99	21.8%
	Yes	299	65.9%
	Maybe	56	12.3%
If yes, at what age are women first invited for BC screening?	Don't know	139	30.6%
	40 years old	315	69.4%
If yes, at what age are women last invited for BC screening?	Don't know	294	64.8%
	75 years	160	35.2%
Have you ever been invited for breast screening on the MOH's Breast Screening Program?	No	261	57.5%
	Yes	132	29.1%
	Maybe	61	13.4%
Have you ever had breast screening on the MOH's Breast Screening Program?	No	278	61.2%
	Yes	115	25.3%
	Maybe	61	13.4%

When it comes to the Ministry of Health's (MOH) BC screening program, it appears that there is a strong awareness with 65.9% of participants being aware of its existence. However, there remains some uncertainty with 30.6% not knowing the age at which women are first invited for screening and a significant 64.8% unaware of when these invitations cease. With regards to actual participation, only 29.1% of participants reported receiving an invitation for breast screening, and a smaller percentage (25.3%) have taken part in the screening (Table 2).

An analysis of associated factors showed that participants with a monthly family income of over 20,000 had significantly higher awareness of BC signs (56.1%). Those who were medically free had a higher level of awareness (45.5%) compared to those with underlying medical conditions. No significant association was found between awareness level and other demographic factors such as age, marital status, educational status, and employment status (Table 3).

**Table 3:** Associated factors with participants' awareness level of BC signs Chi-square and Fisher-Freeman-Halton exact tests *p-value <0.05 BC, breast cancer

n=454		Participant awareness				
		Low awareness		Sufficient awareness		
		N	%	N	%	p-value
Age	≤40	196	62.6%	117	37.4%	0.464
	41-60	73	56.6%	56	43.4%	
	>60	7	58.3%	5	41.7%	
Marital status	Single	96	62.7%	57	37.3%	0.685
	Married	145	59.9%	97	40.1%	
	Divorced	25	55.6%	20	44.4%	
	Widowed	10	71.4%	4	28.6%	
Educational status	No official education	18	60.0%	12	40.0%	0.676
	High school or less	85	63.9%	48	36.1%	
	Bachelor's degree and above	173	59.5%	118	40.5%	
Employment status	No	123	62.1%	75	37.9%	0.629
	Yes	153	59.8%	103	40.2%	
Monthly income of the family (Saudi Riyals)	≤10,000	130	65.3%	69	34.7%	0.013^*^
	10,001-20,000	121	61.1%	77	38.9%	
	>20,000	25	43.9%	32	56.1%	
Medically free	No	144	67.9%	68	32.1%	0.004^*^
	Yes	132	54.5%	110	45.5%	
Diabetes mellitus	No	234	61.1%	149	38.9%	0.792
	Yes	42	59.2%	29	40.8%	
Dyslipidemia	No	248	60.9%	159	39.1%	0.875
	Yes	28	59.6%	19	40.4%	
Hypertension	No	240	60.6%	156	39.4%	0.886
	Yes	36	62.1%	22	37.9%	

When it comes to understanding the risk factors of BC, participants with dyslipidemia had significantly higher awareness (55.3%) compared to those without the condition. However, no significant association was found between the level of awareness and other demographic or health factors (Table 4).

**Table 4:** Associated factors with participants' awareness level of BC risk factors Chi-square and Fisher-Freeman-Halton exact tests *p-value <0.05 BC, breast cancer

n=454		Participant awareness				
		Low awareness		Sufficient awareness		
		N	%	N	%	p-value
Age	≤40	194	62.0%	119	38.0%	0.125
	41-60	67	51.9%	62	48.1%	
	>60	8	66.7%	4	33.3%	
Marital status	Single	92	60.1%	61	39.9%	0.480
	Married	147	60.7%	95	39.3%	
	Divorced	24	53.3%	21	46.7%	
	Widowed	6	42.9%	8	57.1%	
Educational status	No official education	19	63.3%	11	36.7%	0.845
	High school or less	80	60.2%	53	39.8%	
	Bachelor's degree and above	170	58.4%	121	41.6%	
Employment status	No	120	60.6%	78	39.4%	0.631
	Yes	149	58.2%	107	41.8%	
Monthly income of the family (Saudi Riyals)	≤10,000	120	60.3%	79	39.7%	0.722
	10,001-20,000	118	59.6%	80	40.4%	
	>20,000	31	54.4%	26	45.6%	
Medically free	No	127	59.9%	85	40.1%	0.848
	Yes	142	58.7%	100	41.3%	
Dyslipidemia	No	248	60.9%	159	39.1%	0.041^*^
	Yes	21	44.7%	26	55.3%	
Diabetes mellitus	No	232	60.6%	151	39.4%	0.191
	Yes	37	52.1%	34	47.9%	
Hypertension	No	238	60.1%	158	39.9%	0.391
	Yes	31	53.4%	27	46.6%	

## Discussion

The current study was conducted to assess the awareness and predictors of BC screening among females in Saudi Arabia. The findings of the present study showed that almost half of the participants rarely or never checked their breasts. This aligns with El Bcheraoui et al. who also reported that 42.8% of the participants never performed breast self-exam. They further revealed that 25% of participants between the ages of 50 and 74 years had adequate knowledge about breast self-exam [14]. Similar results were shared by Fatima et al. who reported that 43% of women performed self-breast examination regularly [15]. Self-breast examination is a simple, easy-to-perform, and cost-free procedure for the detection of BC. Although there is no strong evidence regarding its impact on reducing mortality, self-breast examination remains an effective approach for promoting awareness in the general public [16]. Othman et al. in their study from Jordan reported that approximately 50% of the respondents performed self-breast examination regularly [17]. A slightly higher prevalence of self-breast examination was reported by AlSaleh et al. who revealed that 57% performed their self-breast examination among Saudi participants [18]. 

Similarly, around one-third of the present study population had a low level of confidence that they would observe changes in their breasts. A study by Qedair et al. reported that 23.7% of their study participants were confident that they would be able to observe changes in their breasts [8]. Generally, there has been a poor level of reported knowledge among the general population regarding the symptoms of BC. A cross-sectional study from Tanzania reported that only 37.7% of the participants were aware of the signs and symptoms of BC [19]. Similarly, another study from India reported that among women who were diagnosed with BC, breast lump was correctly identified by 75% of the study participants whereas a change in the size of the breast was noted by 57% of respondents [20]. Furthermore, almost half of the participants never visited a doctor after noticing a change in their breasts. This is in contrast with the study by Salih et al. who found that 94.6% of the participants in Saudi Arabia had a positive attitude toward consulting doctors if they observed the appearance of BC symptoms [21]. 

The present study also found a high level of awareness regarding the MOH's BC screening program. In the Kingdom of Saudi Arabia (KSA), mammogram screenings (MSs) are provided free of charge to all KSA citizens. The MOH in KSA advises that women aged 40 and above, without a family history of BC, should undergo MS. For those with a family history of BC, screening should begin 10 years earlier than the age of BC onset in their family member. As per MOH policy, women aged 40 to 50 years are recommended to have MS every two years, while those aged 51 to 69 years should undergo screening every one to two years [22].

The findings of the study also showed that participants with higher incomes had significantly higher awareness of BC signs. Previous studies have also reported a link between income education status and knowledge about BC. Several studies have reported that women from low-income backgrounds are less educated and are less aware of BC screening [23,24]. A study by Alsowiyan et al. reported that participants who had an income of more than 5000 riyals had higher awareness levels compared to those below that income level (P=0.012) [25]. 

However, in the present study, no association was found between education status and awareness level of BC. Another significant association identified in the present study was dyslipidemia and BC awareness. The findings showed that more than half of the participants with dyslipidemia had a high awareness level compared to those without dyslipidemia. Some studies have reported that dyslipidemia is a significant risk factor for the poor prognosis of BC [26]. A previous study has reported that dyslipidemia prior to chemotherapy intervention had a negative effect on the complete response rate of BC [27]. The exact role of dyslipidemia in BC remains poorly understood; however, a higher awareness level in this patient population is significant, which warrants further research to elucidate the role of dyslipidemia in BC.

The study possesses several significant strengths. Its cross-sectional design promotes robust data collection and a broad understanding of variables. The diverse participant pool increases the applicability of the results. The structured questionnaire, based on the Breast CAM, ensures precise and relevant data collection. Finally, the comprehensive statistical analysis provides a detailed view of the data, improving the clarity of the results.

This study offers valuable insights into the awareness and predictors of BC screening among Saudi females; however, it has several limitations. First, it was conducted in Jeddah, a major city in Saudi Arabia. The results may not be applicable to females in other parts of Saudi Arabia, especially rural areas where healthcare accessibility and awareness levels could differ. Second, the study relied on self-reported data, which may lead to recall bias. Participants might have overestimated or underestimated their awareness or participation in BC screening programs. Finally, the study's cross-sectional design prevents it from establishing causal relationships between the predictors and awareness of BC screening. Longitudinal studies are necessary to validate the relationships identified in this study.

## Conclusions

In conclusion, this study revealed significant insights into the awareness and associated factors of BC screening among females in Jeddah, Saudi Arabia. The findings underscore the urgent need for targeted interventions to promote regular breast screening, raise confidence in noticing changes in the breasts, and encourage doctor consultations when changes are observed. While the awareness of the MOH's BC screening program was significantly high, the actual participation in the screening was not as robust. This could be due to fear, discomfort, societal norms, or practical issues like time constraints and accessibility. Further research is needed to better understand and address these barriers. This points to the need for further research to understand the gap and devise strategies to increase participation. Income and dyslipidemia emerged as significant associated factors with the awareness of BC signs. Therefore, future interventions should consider these factors to design effective awareness campaigns. Ultimately, enhancing BC screening awareness and practices can contribute significantly to early detection, thereby improving the prognosis and survival rates of BC among Saudi females.
